# Electrochemical Simulation of 25B-NBOMe Phase I Metabolism and Metabolite Profiling by HPLC-QTOF-MS

**DOI:** 10.3390/molecules30224450

**Published:** 2025-11-18

**Authors:** Agata Kot-Wasik, Agnieszka Potęga, Justyna Aszyk-Woźniak, Dorota Garwolińska, Marek Wiergowski, Andrzej Wasik

**Affiliations:** 1 Department of Analytical Chemistry, Faculty of Chemistry, Gdańsk University of Technology, 11/12 Narutowicza St., 80-233 Gdańsk, Poland; agata.kot-wasik@pg.edu.pl; 2 Department of Pharmaceutical Technology and Biochemistry, Faculty of Chemistry, Gdańsk University of Technology, 11/12 Narutowicza St., 80-233 Gdańsk, Poland; agnieszka.potega@pg.edu.pl; 3 Pharmaceutical Plant Polpharma S.A., 19 Pelplińska St., 83-200 Starogard Gdański, Poland; justyna.aszyk-wozniak@polpharma.com (J.A.-W.); dorota.garwolinska@polpharma.com (D.G.); 4 Department of Forensic Medicine, Faculty of Medicine, Medical University of Gdańsk, 23 Dębowa St., 80-204 Gdańsk, Poland; marek.wiergowski@gumed.edu.pl

**Keywords:** 25B-NBOMe, electrochemical simulation of xenobiotic metabolism, on-line electrochemistry-mass spectrometry, phase II metabolites, biological samples analysis, HPLC-Q-TOF-MS, forensic toxicology

## Abstract

This is the first report on the electrochemical simulation of phase I metabolism of 2-(4-bromo-2,5-dimethoxyphenyl)-N-[(2-methoxyphenyl)methyl]ethanamine (25B-NBOMe), a relatively new psychoactive substance available on the illicit drug market. The electrochemical approach enables fast generation and characterization of potential in vivo metabolites, and thus, can assist in the preliminary assessment of xenobiotic activity and toxicity profiles in humans. Phase I oxidation reactions of 25B-NBOMe were simulated in a three-electrode thin-layer electrochemical flow cell. Electrochemically generated products were directly analyzed by high-resolution mass spectrometry. To verify relevance to human metabolism, they were compared with those detected in biological samples taken from individuals severely intoxicated with 25B-NBOMe. The electrochemical conversion of 25B-NBOMe yielded key phase I metabolites—hydroxylated and *N*-desalkylated—along with their corresponding dehydrogenated products. *O*-Desmethylated and bis-*O*,*O*-desmethylated drug derivatives were also formed electrochemically, though in lower amounts. The former was confirmed in gastric contents, blood, and urine samples. Furthermore, phase II metabolites, such as *O*-desmethyl-25B-NBOMe glucuronide and sulfonate, were detected exclusively in some biological specimens, highlighting the complementary role of in vivo analysis. Our findings demonstrate that the electrochemical method provides a promising platform for the rapid and straightforward evaluation of 25B-NBOMe phase I metabolism. The partial overlap with authentic human metabolites supports its relevance as a screening and hypothesis-generating tool. The electrochemical approach, although not fully consistent with data from biological samples, can complement conventional in vitro and in vivo models, aiding in the identification of potential biomarkers and the evaluation of toxicological risk associated with novel psychoactive substances.

## 1. Introduction

In recent years, there has been a huge increase in the number of non-controlled psychoactive substances—known as “designer drugs”—that are constantly appearing on the illicit drug market [1,2,3]. Among them, a new class of synthetic hallucinogenic N-2-methoxybenzyl-substituted phenethylamine drugs, commonly referred to as “NBOMes”, has become widely distributed. The most frequently reported drugs in this group are 25I-, 25B-, 25N-, and 25C-NBOMe [4,5,6]. In humans, NBOMe compounds are usually active at sub-milligram doses and interact with various neurotransmitter systems, acting especially as highly efficacious brain serotonin 5-HT2A and 5-HT2C receptor agonists [7,8]. Numerous reports have been published on cases of toxicity and multiple fatalities resulting from the consumption of NBOMes [9,10,11]. Therefore, their rapid spread around the world may pose a threat to public health, particularly as it relates to the health of young people. Knowledge of the metabolic fate of a drug in the body is essential for forensic and toxicological investigations. However, the available scientific evidence concerning the metabolism of NBOMe designer drugs in humans, to predict potential complications and drug interactions, is still very limited. Generally, little has been reported on the targeted oxidation (phase I metabolism) and/or conjugation (phase II metabolism), and subsequent mass spectrometric (MS) detection of psychoactive substances, doping agents, or related compounds with the aim of metabolite prediction and identification [12,13,14]. It seems somewhat astonishing, considering that most target compounds carry electroactive functional groups that can be susceptible to oxidation-reduction (redox) reactions under appropriate conditions. Such conditions can be created by electrochemical methods, which, in general, have been successfully implemented in environmental, forensic, and life science applications [15,16,17]. As the analysis of NBOMes and their metabolites in biological materials can be challenging, special emphasis is given to the development of modern sensitive analytical techniques that can generate and identify the variety of drug metabolites that are also being formed in vivo.

The main route of drug elimination is biotransformation, occurring due to various enzymes (i.e., drug-metabolizing enzymes) present in the body. Enzymatic biotransformation is usually initiated by oxidation reactions (phase I metabolism) involving enzymes of the cytochrome P450 (P450) family. In in vitro practice, these originate from the liver, including an isolated perfused animal liver, liver slices, hepatocytes, or, most importantly, liver-cell microsomes [18,19,20], while in vivo experiments involve laboratory animals [20]. However, performing these biological schemes is usually laborious, time-consuming, and of limited reproducibility [21]. As an alternative to the above conventional methods for studying enzymatic drug biotransformation, the electrochemical simulation of P450-mediated oxidation reactions has been developed [22,23,24]. Electrochemistry (EC), as a purely instrumental technique, enables the formation of drug products in the absence of biological matrices in the reaction medium in a very short time. This allows for overcoming many of the laborious tasks associated with the isolation and further identification of metabolic products formed in vitro (hepatocytes, liver-cell microsomes, purified enzymes, etc.) or in vivo (urine, blood, etc.) [23,25,26]. Thus, the application of EC is considered to be a promising time-saving and cost-effective approach that improves the conventional methods of drug metabolism studies. The combination of EC coupled on-line to analytical techniques such as MS with or without previous liquid chromatographic separation (EC-LC-MS or EC-MS) creates a rapid and convenient platform for the generation (in the electrochemical flow-through cell) and detection (by MS and/or LC) of a series of drug oxidation products, which usually show a good correlation with metabolites found in vivo [27,28]. Additionally, EC has been utilized in combination with powerful detection techniques other than MS, particularly with nuclear magnetic resonance (NMR) spectroscopy [29,30], which allows full characterization of the electrochemical products. These would be useful for obtaining reference standards of the parent drug metabolites, which are often unavailable in many laboratories. The importance of EC-(LC-)MS hybrid techniques is emphasized by a number of reviews that have been published recently [31,32,33]. These also seem to have the greatest potential of being applied in future toxicological and forensic applications.

**Figure 1:**
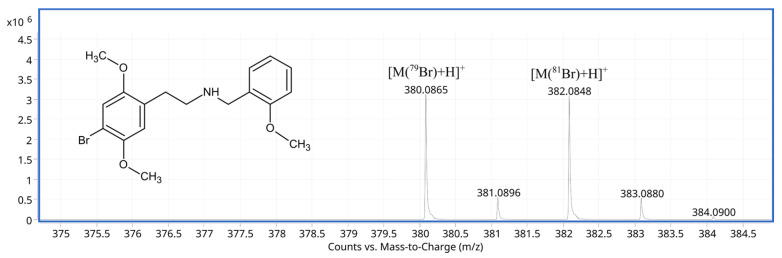
Structure of 25B-NBOMe (2-(4-bromo-2,5-dimethoxyphenyl)-N-[(2-methoxyphenyl)methyl]ethanamine) and its representative mass spectrum revealing a characteristic isotopic pattern.

Knowledge of the metabolic pathway(s) of NBOMe designer drugs is crucial for the elucidation of their possible toxicity and mechanism of action in the human body. Previous studies on the enzymatic biotransformation of NBOMes have been performed using incubation with human and rat liver microsomes (HLMs and RLMs, respectively) or in human and rat urine, followed by LC-high-resolution MS analysis. According to recently published data, NBOMe drugs are extensively metabolized, in particular through P450 and uridine 5′-diphospho-glucuronosyltransferase (UGT) enzymes. The primary metabolic pathways of NBOMes, such as 25I-, 25B-, 25N-, and 25C-NBOMe, include mainly: *O*-desmethylation, followed by bis-*O*,*O*-desmethylation and hydroxylation, and N-desalkylation, as well as extensive glucuronidation and sulfation of the major phase I metabolites [12,13,34,35,36]. It is also known that in all the studied cases, hydroxylation preferentially occurs on the NBOMe ring. However, the identification and quantification of 25B-NBOMe (2-(4-bromo-2,5-dimethoxyphenyl)-N-[(2-methoxyphenyl)methyl]ethanamine, Figure 1)—a popular recreational drug—and its metabolic products have become a significant analytical challenge. This is primarily due to the compound’s extreme potency and the fact that its metabolites are often difficult to detect or quantify using standard, cost-effective toxicological screening methods typically employed in forensic laboratories.

Further, the rapid transience of the NBOMes on the illicit drug market, the initial lack of analytical reference standards, and many possible structures are still very challenging for forensic and clinical toxicology [37]. Screening for their metabolites with the use of high-performance LC coupled to quadrupole time-of-flight mass spectrometry (HPLC-Q-TOF-MS) and tandem mass spectrometry (HPLC-Q-TOF-MS/MS) has become nowadays the technique of choice for many toxicological investigations [38]. Moreover, it should also be noted that these compounds are not amenable to gas chromatography-MS analysis due to their thermolabile and hydrophilic properties. Thus, the available NBOMes metabolism data are still insufficient. The simplicity of the electrochemical system, combined with its ease and speed of application to a large number of compounds, makes it a valuable tool in drug metabolism research. Much of the more recent work in this regard has been carried out by Mielczarek et al. [39] and other authors, including Jerszyńska et al. [40] and Smoluch et al. [41], who have used electrochemistry to investigate substances such as cocaine, methamphetamine, dextromethorphan, and benzydamine. To the best of our knowledge, no such data exists for 25B-NBOMe and any of its related compounds.

The aim of the study was to evaluate applicability of an electrochemical method to simulate the metabolism of the designer drug 25B-NBOMe into phase I metabolites. The electrochemically generated oxidation products of the drug standard solution were structurally characterized based on accurate mass analysis and MS/MS fragmentation patterns, and subsequently compared with metabolites detected in biological samples from real severe intoxication cases. Such integrative analytical approach provides a rapid, ethical and instrument-based alternative to conventional in vivo/in vitro models offering a convenient understanding of 25B-NBOMe biotransformation pathways in the human body.

## 2. Results and Discussion

Although the psychoactive substances of the NBOMe group have been known for over a decade, very little toxicological data, both clinical and forensic, concerning 25B-NBOMe has been published so far. The metabolism of this relatively new designer drug and how it is broken down by drug-metabolizing enzymes into various metabolites in the human body are still not fully understood [42]. Phase I and II metabolites of this compound were first detected in an untargeted screening, followed by liquid chromatography-tandem mass spectrometry (LC-MS/MS). In order to clarify the major metabolic pathways of 25B-NBOMe and to improve its metabolite detection window in biological matrices, Boumrah et al. [43] conducted in vitro studies using human liver microsomes (HLMs). The MetaboLynx™ algorithm—an important structure elucidation tool applied by this research group—indicated that the 25B-NBOMe biotransformation included hydroxylation, *O*-desmethylation, N-desalkylation, dehydrogenation, and combinations thereof. The hydroxylated metabolite was the most abundant compound after the phase I process. Thus, the two major metabolic pathways of the drug in humans appear to be the cytochrome P450 (P450) oxidative metabolism and UGT-mediated glucuronidation. The sulfate conjugation of 25B-NBOMe by sulfotransferases (SULT) is also possible. These results provided helpful information establishing valuable biomarkers in the case of 25B-NBOMe ingestion.

The demand for reference standards of drug metabolites has driven the development of innovative techniques for simulating drug metabolism. EC has emerged as a valuable tool for predicting metabolic pathways across a wide range of chemical classes. An experiment using the ROXY™ EC system in on-line combination with high-resolution MS (a Q-TOF), presented in this paper, is a substitute for in vivo studies—it is designed to mimic enzymatic redox reactions that occur in nature and/or to elucidate the structure of metabolites that are difficult to characterize in biological matrices [33,44]. Therefore, its application should be able to reproduce what happens in the body of a person who has taken psychoactive substances, in this case, 25B-NBOMe. Theoretically, the results obtained from the electrochemical conversion of 25B-NBOMe should be largely consistent with those obtained by analyzing the drug with drug-metabolizing enzymes from liver subfractions (e.g., HLMs) or from various biological materials by HPLC coupled to a Q-TOF mass analyzer [15,28,40].

### 2.1. Optimization of Experimental Conditions of 25B-NBOMe Electrochemical Conversion

Optimization of the conditions for the electrochemical conversion of 25B-NBOMe included several parameters such as flow rate through the thin-layer electrochemical ReactorCell™, analyte concentration, pH, and ionic strength of the electrolyte. A One-factor-at-a-time (OFAT) approach has been taken until satisfactory results in terms of the number, signal intensity and spectra quality for known 25B-NBOMe metabolites were achieved. The threshold for the acceptance of the spectra was set to 20 times noise level. Finally, the electrochemical process was found to work well in a 0.1% aqueous solution of FA and MeOH as a modifier (1:1, *v*/*v*) with a sample solution flow rate at 10 μL/min. Studies carried out in an alkaline environment did not give the expected results (a lack of most 25B-NBOMe metabolites). The best efficiency of electrochemical conversion of 25B-NBOMe into its expected oxidation products was achieved using a GC working electrode and the scan mode in the potential range of 0–2500 mV with incremental steps of 10 mV/s (Table 1).

### 2.2. Results of Electrochemical Simulation of Phase I Metabolism of 25B-NBOMe

**Figure 2:**
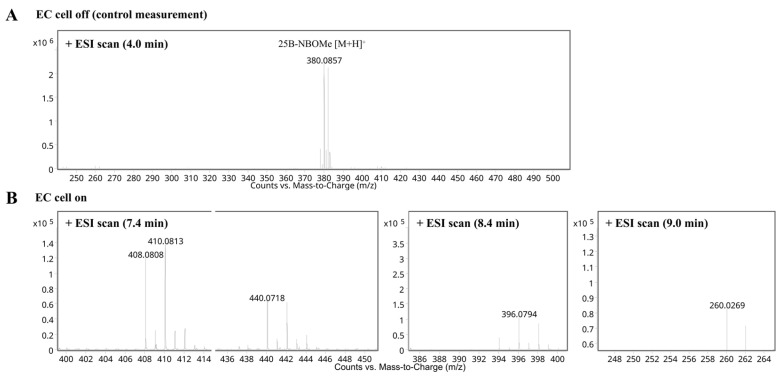
The representative mass spectra of 25B-NBOMe (*m*/*z* 380.0857) and selected EC reaction products (**A**) before (EC cell off) and (**B**) after (EC cell on) the reaction in the thin-layer electrochemical flow cell at various times of measurement (positive ion mode).

The purpose of 25B-NBOMe oxidation in the ROXY™ EC system was to generate products of this psychoactive drug in a matrix-free environment and then to confirm the identification of their presence by HPLC-(ESI)-Q-TOF-MS in samples of biological materials taken from people who had ingested the substance. This approach could close the uncertainty gap and, with the right solutions, enable the laboratory to improve confidence in its results. The electrochemical simulation of 25B-NBOMe oxidative (phase I) metabolism was performed under pre-determined experimental conditions. 25B-NBOMe was initially observed in full scan in both positive and negative ion modes; however, the positive mode was ultimately used for further investigation as it showed better sensitivity and specificity for the parent compound and its products. Bromine-containing molecules, such as 25B-NBOMe, exhibit a distinct isotopic pattern, characterized by the predominance of two primary bromine isotopes: ^79^Br (50.69%) and ^81^Br (49.31%) [45]. This characteristic isotopic pattern (Figure 1) is crucial for identifying brominated compounds during MS analysis. The expected molecular ion at *m*/*z* 380.0857 relevant to the protonated target psychoactive drug was observed in the mass spectrum recorded in the absence of electrochemical potential (EC cell off; control measurement) (Figure 2A). A notable drop in the abundance of the parent molecule signal was noticed when the electrode potential was applied (EC cell on). This change was attributed to the oxidation of 25B-NBOMe into several potential phase I products (Figure 2B) in a thin-layer electrochemical ReactorCell™.

**Figure 3:**
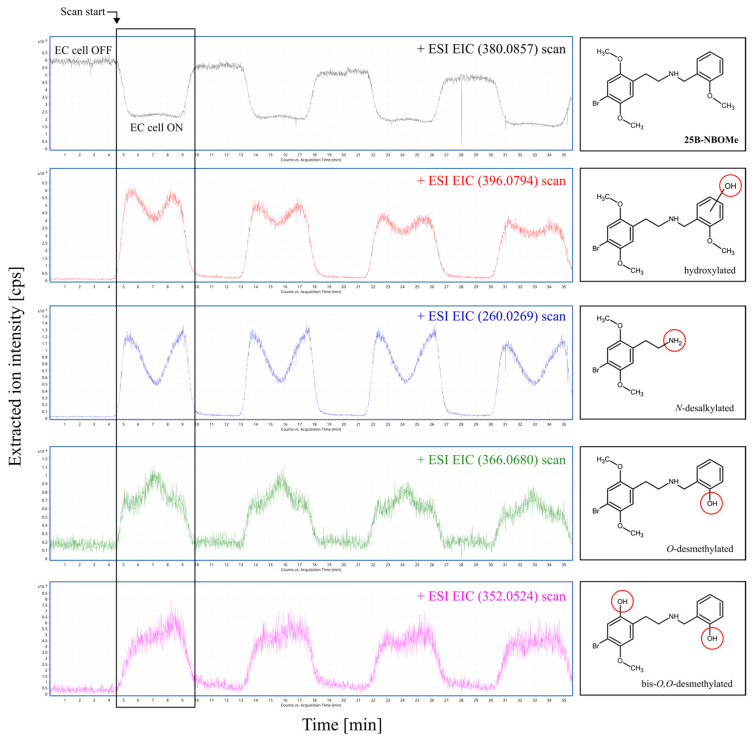
Extracted ion chromatograms (EICs) obtained after 10 μM 25B-NBOMe oxidation at a disc GC working electrode (positive ion mode). The *m*/*z* ratios shown correspond to the protonated molecular ion [M+H]^+^ of 25B-NBOMe and its main phase I metabolic products with proposed structures. Experimental conditions: working electrode potential range 0–2500 mV vs. Pd/H_2_; scan rate 10 mV/s, continuous; room temperature; GC working electrode diameter 8 mm.

Two distinct groups of 25B-NBOMe products were obtained during the electrochemical process. The first group consisted of drug derivatives that were formed directly at the GC working electrode surface through electrochemical reactions, likely resulting from oxidation or structural modifications of the parent compound under the applied electrochemical conditions. The formation of various 25B-NBOMe products depended on the working electrode potential. The extracted ion chromatograms (EICs) were generated for specific *m*/*z* values corresponding to spectral peaks recorded during EC-MS analyses (Figure 3). The putative structures of the compounds were deduced based on the (ESI)-Q-TOF-MS experiments and further supported by data from in vitro drug metabolism studies reported in the literature [34,35,38].

Another type of follow-up reaction observed in the electrochemical system was dehydrogenation, which involved the removal of two hydrogen atoms (–2H) from either 25B-NBOMe or its intermediate species through electron transfer processes. This transformation likely led to the formation of unsaturated or more oxidized products, indicated by ions at *m*/*z* 378.0699, 394.0649, and 258.0124, suggesting additional structural modifications beyond the initial oxidation step. However, the interpretation of mass spectra was complicated by the overlapping bromine isotopic patterns of the base compounds and their respective dehydrogenated products, making it difficult to distinguish between them clearly. Consequently, the use of a high-resolution mass spectrometer with a mass resolution exceeding 100,000—such as an Orbitrap mass analyzer—is essential for obtaining sufficiently accurate mass determinations and for resolving observed isotopic interferences, thereby enhancing the overall reliability of the data.

The second group of 25B-NBOMe products appeared to originate from secondary reactions between intermediate species and components of the electrolyte. Specifically, we observed carbonylation (formylation) reactions, in which a formyl group (–CHO), most likely derived from formic acid present in the electrolyte solution, was introduced into the molecular structure. This was evidenced by the presence of ions with *m*/*z* of 408.0808 and 440.0710. These follow-up reactions suggested that the electrolyte not only facilitated the electrochemical process but also actively contributed to further chemical transformations. This expands the diversity of the final product mixture.

A summary of the main electrochemically generated products of 25B-NBOMe is given in Table 2. The experimentally measured *m*/*z* values of the products formed showed good agreement with their respective theoretical *m*/*z* values, with the absolute mass accuracy not exceeding a threshold of 5.5 ppm. Minor discrepancies may be attributed to the instrument’s resolution, non-perfect tuning and/or natural isotopic shifts in the mass spectra.

### 2.3. Detection and Tentative Identification of 25B-NBOMe Metabolites in Biological Samples of Severe Intoxications

With a view to investigating the metabolic pathway of 25B-NBOMe in humans, the data originating from biological samples obtained from patients who had consumed 25B-NBOMe were also studied in detail. The presence of the drug and its potential metabolites in gastric contents, blood, and urine was confirmed by HPLC analyses and mass spectra. Gastric contents, although not subject to phase I and/or II metabolism, were analysed to confirm that 25B-NBOMe had been ingested rather than administrated by injection. These results were compared with the results obtained from the proposed EC-MS method with the use of an electrochemical cell for the generation of 25B-NBOMe oxidation products.

**Figure 4:**
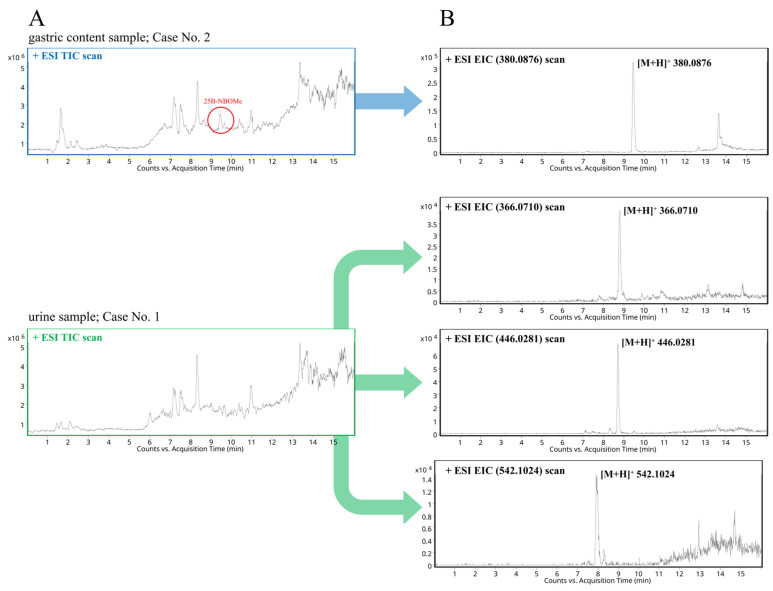
(**A**) The representative total ion chromatograms (TICs) obtained after chromatographic separation of gastric contents and urine samples collected from the deceased patients (Case No. 2 and Case No. 1, respectively) following oral administration of 25B-NBOMe and (**B**) the representative extracted ion chromatograms (EICs) obtained after pseudo molecular ion extraction for 25B-NBOMe and its relevant phase I and II metabolites.

The representative total ion chromatograms (TICs) of gastric contents and urine samples collected from the dead patients (Case No. 2 and Case No. 1, respectively) after oral intake of 25B-NBOMe are shown in Figure 4A. By application of the HPLC-(ESI)-Q-TOF analysis, the parent drug and its several known phase I and II metabolites were successfully identified, and these are characterized in Table 3. The pseudo molecular ions corresponding to the psychoactive drug and its expected metabolites were extracted from the TICs, and respective EICs are shown in Figure 4B.

For the identification of 25B-NBOMe and its metabolites in authentic biological samples, several analytical criteria were applied, including a mass accuracy threshold of ± 5.5 ppm (similar to EC-MS) and the presence of an isotopic pattern characteristic of the bromine compound. Elemental composition analysis was performed using Mass Hunter Workstation Qualitative Analysis, B.03.01 Software (Agilent Technologies, Santa Clara, CA, USA), based on the accurate mass of the protonated precursor monoisotopic ion of *m*/*z* 380.0855 (t_R_ = 9.45 min). Within these specific parameters, only one molecular formula—[C_18_H_23_BrNO_3_]+-was found to match. Subsequent MS/MS fragmentation of the *m*/*z* 380.0855 ion provided two major product ions at *m*/*z* 121.0112 and *m*/*z* 91.0544. Identification of particular compounds was further supported through comparison with fragmentation spectra from commercially available NIST 2.0 Software (Adaptas Scientific Instrument Services, Palmer, MA, USA) with an in-house reference database containing 885 compounds of forensic relevance. Database searches provided a single structural match for each elemental composition.

An unknown compound with precursor ion at *m*/*z* 380.0876 was thus identified as 25B-NBOMe. Additionally, the characteristic bromine isotopic pattern at 79 (M) and 81 (M + 2) in 50.69% and 49.31% abundance, respectively [45], confirmed the presence of 25B-NBOMe in real human biological samples. Final confirmation of the presence of 25B-NBOMe in biological samples was achieved by comparison of the mass spectra and retention times obtained from the analyte detected in blood samples with those of the commercially available 25B-NBOMe reference standard. The retention time of the standard of 25B-NBOMe (9.50 min) matched well with that observed in the blood samples. To additionally verify the compound’s identity, MS/MS analysis was performed. Product ion spectra obtained from both the standard and the blood sample at the same collision energy (~25 V) showed the same fragmentation patterns, finally confirming the structural identity of the compound.

**Figure 5:**
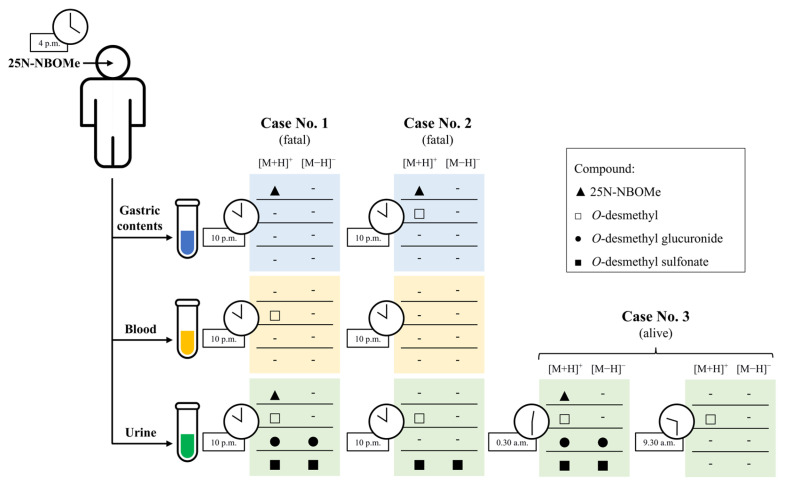
Summary of all relevant compounds identified in human biological materials with HPLC-(ESI)-Q-TOF-MS (both positive and negative ion modes) analysis.

Similarly, 25B-NBOMe and its major metabolites were identified in both urine and gastric contents samples. Identification of the compound was confirmed by the detection of several metabolites displaying the typical isotopic pattern of bromine and a distinct set of MS/MS fragment ions (Table 4). On the basis of these findings, we can conclude that 25B-NBOMe is extensively metabolized, primarily through *O*-desmethylation followed by phase II conjugation via glucuronidation and sulfonation (Figure 5). This metabolic profile is consistent with recently published analyses regarding NBOMes and their metabolites [12,34,35,37]. The appearance of glucuronidation products was revealed in blood and urine samples; in turn, a sulfonation product was noticeable only in urine samples.

The enzyme-catalyzed chemical transformations of drugs in the body usually occur in two biotransformation phases (phase I and phase II). Based on the results presented, it can be predicted that in humans, 25B-NBOMe is rapidly absorbed from the gastrointestinal tract and further metabolized through an oxidative pathway in the liver.

**Figure 6:**
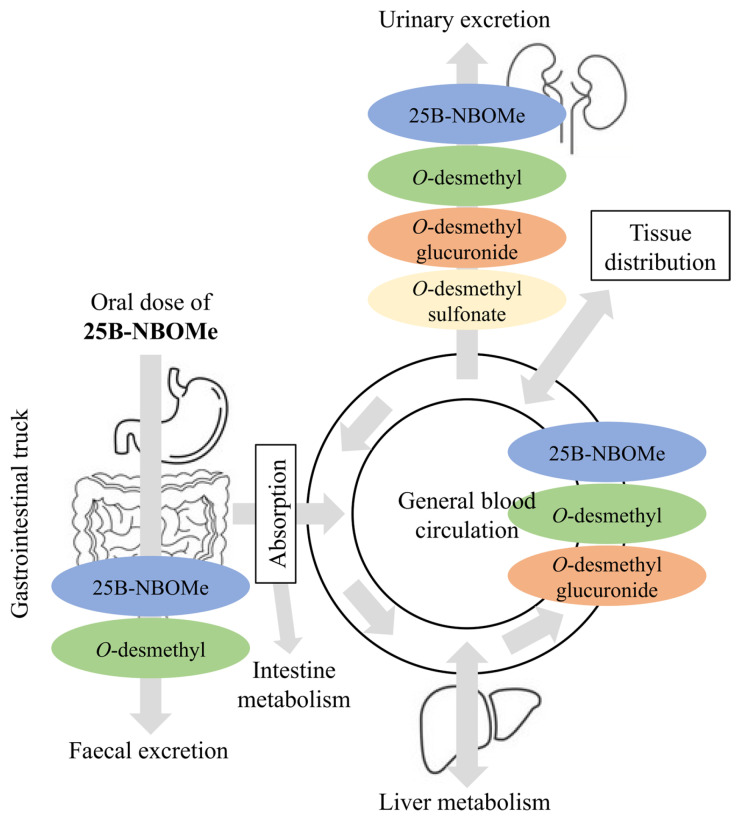
Schematic representation of the proposed 25B-NBOMe metabolic transformation in the human body after oral drug administration.

Furthermore, the liver is the primary site of drug metabolism through desmethylation, a reaction where a methyl group is removed from the molecule [46]. Consequently, three distinct *O*-desmethylated metabolites of 25B-NBOMe are possible, followed by glucuronide conjugate formation, a metabolic route commonly observed with other NBOMes [12,13,34,35]. Subsequently, it was found that the glucuronide conjugates of the compound 25B-NBOMe itself or its *O*-desmethylated and hydroxylated derivatives do not cross the blood–brain barrier [35]. There is solid evidence that the drug’s toxic effects are caused solely by the glucuronide metabolites, which are more abundant in plasma than 25B-NBOMe itself following the ingestion of a pharmacological dose [34]. Therefore, these may be prime candidates for reliable biomarkers in the forensic identification of 25B-NBOMe intoxication. Additionally, hydroxylation and N-desalkylation, which result in the loss of the methoxybenzyl group, may occur. The parent drug and its most abundant metabolites are also excreted in the urine, where they are extensively conjugated with sulfate. A schematic representation of the proposed metabolic transformations of 25B-NBOMe in the human body after oral administration is shown in Figure 6.

**Figure 7:**
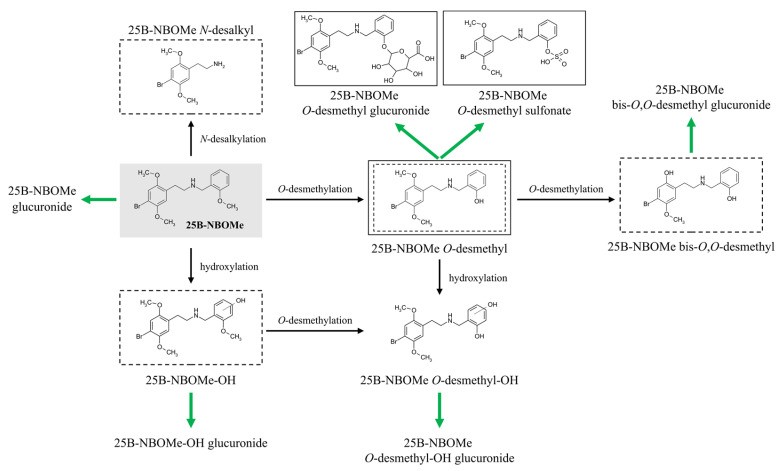
A general overview of the major metabolic pathways of 25B-NBOMe in the human body. The products generated after electrochemical 25B-NBOMe oxidation and directly detectable in human biological materials are marked in the frames (dashed and solid lines, respectively). Black arrows—products of the phase I metabolism, green arrows—products of the phase II metabolism. The scheme was developed based on [43].

Generally, the major metabolic pathways of 25B-NBOMe described within this study included mono- and bis-*O*,*O*-desmethylation, hydroxylation at various positions (affecting either the benzyl (NBOMe) or phenyl ring), N-desalkylation, and combinations thereof, followed by glucuronidation and/or sulfation of primary metabolites (Figure 7). These findings are in good accordance with the existing literature. Notably, the same potential phase I metabolite (O-desmethylated) was detected both after electrochemical oxidation of 25B-NBOMe and in human biological samples. Thus, this correspondence demonstrates that the electrochemical simulation effectively mirrors of metabolic transformations occurring in the human body following ingestion of this psychoactive drug. Once more, it becomes evident that EC-(LC-)MS hybrid techniques seem to have great potential to be applied in future toxicological and forensic applications with emphasis on metabolic and mechanistic investigations of electrochemical reactions occurring in various contexts. The information obtained on the electrooxidation products of 25B-NBOMe is important for a general understanding of the toxicity of NBOMes and for identifying metabolites that could serve as valuable biomarkers of drug poisoning.

However, it is important to note that while the electrochemical method offers a rapid, informative, and solvent-minimized approach to simulating xenobiotic oxidative metabolism, it also has inherent limitations. Unlike enzymatic systems, it lacks biological specificity and does not replicate enzyme-substrate interactions or the stereoselectivity characteristic of cytochrome P450-mediated reactions.

Moreover, electrochemically generated products may include artifacts or over-oxidized species that do not occur in vivo. Therefore, EC-based systems should be regarded as complementary tools rather than replacements for conventional in vitro or in vivo metabolism studies.

## 3. Materials and Methods

### 3.1. Chemicals and Biological Materials

Acetonitrile HPLC gradient (ACN), formic acid (FA), and methanol HPLC gradient (MeOH) were purchased from Merck KGaA (Darmstadt, Germany). Standard 25B-NBOMe was obtained from Cayman Chemical Company (Ann Arbor, MI, USA). Internal standard racemic methamphetamine-d5 (rac-Meth-d5) was purchased from LGC Standards (Luckenwalde, Germany). All other chemicals and solvents were of the highest quality commercially available. Deionized water (conductivity of <0.055 μS/cm), used in all the experiments, was produced in an HLP5 system with a 0.2 μm capsule filter (Hydrolab Sp. z o.o. Sp.K., Straszyn, Poland).

A stock solution of 25B-NBOMe was prepared in a mixture of ACN and water (1:1, *v*/*v*) at a concentration of 100 ng/mL. A stock solution of rac-Meth-d5 was prepared as above at a concentration of 1 µg/mL, then diluted to 5 ng/mL and used for all analyses. Both stock solutions have been stored at –20 °C until they were used.

Post-mortem specimens (gastric contents, blood, and urine) were collected from two dead young persons (signed as Case No. 1 and Case No. 2) during their autopsies performed within 24 h after the death. Blood and urine samples were also collected from an alive patient (signed as Case No. 3) during hospitalization (6 and 15 h after designer drug ingestion). Biological samples have been stored at −80 °C until analysis.

### 3.2. Untargeted Analysis

#### 3.2.1. Electrochemical Simulation of Phase I Metabolism of 25B-NBOMe

For electrochemical metabolism simulation, a stock solution of 25B-NBOMe was dissolved in an electrolyte solution (a mixture of MeOH and water (1:1, *v*/*v*) with 0.1% FA) to reach a concentration of 10 μM. Simulation of phase I oxidation reactions of 10 μM 25B-NBOMe was accomplished at a constant flow rate of 10 µL/min in a three-electrode thin-layer electrochemical flow cell (electrochemical reactor cell, the ReactorCell™, EC cell) from Antec Leyden (Zoeterwoude, The Netherlands). For the continuous synthesis of specific drug metabolites, the working electrode potential was swept from 0 to +2500 mV with incremental steps of 10 mV/s, and was controlled by Dialogue software (p/n 210.9005, Antec Leyden, Alphen Aan Den Rijn, The Netherlands). The outlet of the EC cell was directly connected to the electrospray ionization (ESI)-Q-TOF-MS source. To register mass intensity-potential curves, the continuous scan mode was used. Representative results of at least three independent experiments were considered. The relevant EC parameters are presented in Table 1. EC experiments were performed without chromatographic separation to reduce the complexity of the system.

#### 3.2.2. Screening of 25B-NBOMe Metabolites Present in Human Biological Materials

**Figure 8:**
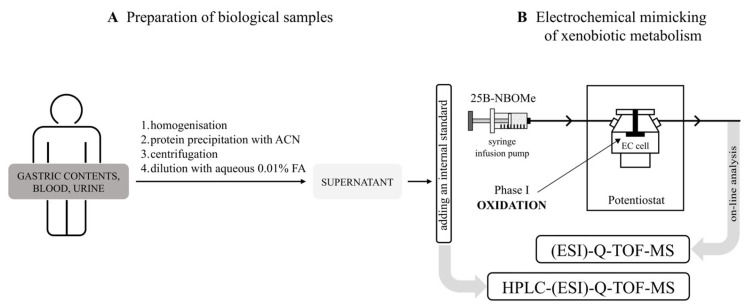
Workflow and system for simulation and analysis of 25B-NBOMe metabolism. (**A**) Presentation of the sample preparation steps from various biological materials for HPLC-(ESI)-Q-TOF-MS analysis and (**B**) schematic representation of the system used for simulation of phase I metabolism of 25B-NBOMe in a three-electrode thin-layer electrochemical flow cell (EC cell) with on-line (ESI)-Q-TOF-MS analysis.

Fast screening of 25B-NBOMe metabolites present in biological materials derived from patients severely intoxicated with the drug was carried out in scan mode by an HPLC-(ESI)-Q-TOF-MS. For this purpose, gastric contents, blood, and urine samples were prepared using a simple protein precipitation method with ACN. Briefly, 200 µL of the biological material was transferred to a 1.5 mL Eppendorf vial, followed by the addition of 400 µL of ACN. The mixture was then centrifuged at 7000 rpm for 5 min, allowing for the separation of proteins and other cellular components. Subsequently, 400 µL of the resulting supernatant was diluted 1:1 (*v*/*v*) with water containing 0.01% FA. Further details on the sample preparation protocols can be found in the study by Wiergowski et al. [38]. A visual comparison of the sample preparation steps from various biological materials is shown in Figure 8A. To enhance the accuracy of the procedure, 10 µL of the internal standard (rac-Meth-d5 in MeOH) at a concentration of 1 µg/mL was used. The final mixture was transferred to an autosampler vial and analyzed by HPLC-(ESI)-Q-TOF-MS.

### 3.3. General Instrumentation

#### 3.3.1. Mimicking of Phase I Metabolism-EC Conditions

The system used for electrochemical simulation of phase I metabolism of 25B-NBOMe (Figure 8B) contained a commercial thin-layer electrochemical ReactorCell™ (EC cell) equipped with a disc glassy carbon (GC) working electrode (f = 8 mm; A = 0.502 cm^2^), the HyREF™ palladium–hydrogen (Pd/H_2_) reference electrode, and an upper part of the inlet block made of the carbon-loaded polytetrafluoroethylene (PTFE) as an auxiliary electrode (a three-electrode configuration) from Antec Leyden. Glassy carbon is widely used as an electrode material in electrochemistry due to its wide potential window, good electrical conductivity, high resistance to chemical attack, and impermeability to gases and liquids. Electrochemical measurements were carried out via the ROXY™ potentiostat driven by Dialogue software (Antec Leyden). The sample solutions for electrochemical analysis were infused through the EC system using an SP2-ROXY™ Dual-Piston Syringe Pump (Antec Leyden).

In the event of loss of sensitivity due to fouling or compound absorption, the surface of the GC working electrode was wiped with a tissue wetted with MeOH and/or polished with a polishing disc and diamond slurry provided by the manufacturer (Antec Leyden) prior to each experiment.

The outlet of the EC cell was connected directly to a dual ESI source of a 6560 Ion Mobility LC-Q-TOF mass spectrometer (Agilent Technologies, Santa Clara, CA, USA) using a 50 cm long PEEK tubing (130 µm I.D.). ESI-MS detection of products generated in the EC cell was performed in both positive and negative ion modes in the full scan MS and MS/MS (mass-to-charge ratio, *m*/*z* 50–1000). The ESI-MS was operated with the following parameters: fragmentor voltage at 320 V, nebulizer gas (N_2_) pressure at 35 psig, capillary voltage at 4000 V, nozzle voltage at 2000 V, and drying gas (N_2_) flow rate and temperature were set to 10 L/min and 350 °C, respectively.

#### 3.3.2. Qualitative Analysis-HPLC-(ESI)-Q-TOF-MS Conditions

The HPLC-(ESI)-Q-TOF-MS was performed with the use of an Agilent 1290 LC system equipped with a binary pump, an on-line degasser, an autosampler, and a thermostatted column compartment coupled to a 6540 Q-TOF-MS with a Dual ESI source (Agilent Technologies). An Ascentis Express C18 (100 × 4.6 mm, 2.7 μm particle size) (Supelco Inc., Bellefonte, PA, USA) or a Kinetex C18 (150 × 2.1 mm, 1.7 μm particle size) (Phenomenex Inc., Torrance, CA, USA) column was used for the reversed-phase HPLC separation.

The mobile phase consisted of water containing 0.01% *v*/*v* of FA (component A) and ACN containing 0.01% *v*/*v* of FA (component B). The following gradient elution program was applied: 0–2 min (20% B), 2–12 min (60% B), 12–15 min (60% B), 15.01 (20% B), and 15.01–19 min (20% B). The column temperature throughout the separation process was kept at 40 °C. The mobile phase flow rate was 0.3 mL/min and the injection volume was 10 µL. During the analysis, the samples were kept in an autosampler at 4 °C. The ESI source was operated in both positive and negative ion modes with the following conditions: the fragmentor voltage was set at 120 V, nebulizer gas (N_2_) pressure was set at 35 psig, the capillary voltage was set at 3500 V, and drying gas (N_2_) flow rate and temperature were set at 10 L/min and 325 °C, respectively. Data were acquired in centroid and profile modes using the high-resolution mode (4 GHz). The mass range was set at *m*/*z* 50–1000 in MS and MS/MS modes.

All MS data were processed with the Mass Hunter Workstation Qualitative Analysis, B.03.01 Software (Agilent Technologies). The Q-TOF-MS instrument was calibrated daily to ensure optimal performance.

## 4. Conclusions

Our knowledge of the biological background of psychoactive drug metabolism is continually expanding; however, many mechanisms remain unknown or insufficiently explained. A thorough understanding of their metabolic pathways is essential for developing comprehensive screening procedures and improving both forensic and clinical toxicology diagnostics. Progress in this area of research critically depends on the advancement of methods for the generation and identification of various types of drug metabolites.

The present study aimed to compare the metabolic profile of 25B-NBOMe—used as a model compound—by analyzing real biological samples from cases of severe intoxication and by applying an electrochemical simulation of phase I drug metabolism. This latter approach is considered a fast and convenient tool for investigating drug metabolic transformations and, to the best of our knowledge, has not yet been applied to compounds from the NBOMe group.

The electrochemical oxidation of 25B-NBOMe led to the formation of four major phase I metabolites previously described in the literature. In addition, secondary products such as dehydrogenated and carbonylated derivatives were observed. Among the electrochemically generated compounds, only the *O*-desmethylated product corresponded to a metabolite identified in authentic human samples. The highest number of phase II metabolites, including *O*-desmethyl-25B-NBOMe glucuronide and sulfonate, was detected exclusively in the biological model.

Although EC-based metabolism model does not fully reflect the enzymatic specificity of biological systems, the electrochemical approach demonstrates significant potential as a rapid and cost-effective method for simulating drug metabolic pathways, particularly in early-phase toxicokinetic investigations or when conventional in vitro and in vivo approaches are not feasible. As a novel and an increasingly recognized technique, electrochemistry provides unique insights into metabolic transformations—especially those involving short-lived intermediates. The further development of integrated techniques, such as EC-MS, could enable more sensitive and precise analysis of psychoactive substances and their metabolites. To fully incorporate electrochemical techniques into routine toxicological assessments, further validation and quantitative studies on the toxicity of identified metabolites will be essential.

In conclusion, this study highlights the potential of electrochemistry (EC) as a promising tool for elucidating the toxicokinetics of NBOMe compounds in future research. The identification of *O*-desmethylated and conjugated metabolites in real human samples supports their relevance as potential biomarkers for the screening and confirmation of severe intoxications. The integration of EC with computational approaches may further enhance predictive toxicology, contributing to a more comprehensive understanding of the risks and effects on human health associated with NBOMe use. Although further validation using larger cohorts and quantitative protocols is necessary, this strategy may complement traditional in vitro and in vivo models, offering a practical and cost-effective addition to forensic and clinical toxicology laboratories dealing with novel psychoactive substances.

## Figures and Tables

**Table 1:** The electrochemical parameters that were applied to mimic the phase I metabolism of 25B-NBOMe in a three-electrode thin-layer electrochemical flow cell (EC cell).

	Parameter	Value/Characteristic
EC setting	Flow rate	10 µL/min
	Working electrode potential	0–2500 mV (10 mV steps)
	EC operating mode	Scan, continuous
	EC temperature	Room, not controlled

**Table 2:** An overview of the electrotransformation products of 25B-NBOMe that were generated by the EC and identified by the MS.

Measured *m*/*z* ^a^	Theoretical *m*/*z* ^b^	Relative Mass Accuracy [ppm]	Ion Formula	Modification	Metabolic Reaction (Phase I)
380.0857	380.08558	0.32	[C_18_H_23_BrNO_3_]^+^	[M+H]^+^	(parent compound)
378.0699	378.06993	−0.08	[C_18_H_21_BrNO_3_]^+^	[M-2H+H]^+^	dehydrogenation
396.0794	396.08050	−2.78	[C_18_H_23_BrNO_4_]^+^	[M+O+H]^+^	hydroxylation
394.0649	394.06485	0.13	[C_18_H_21_BrNO_4_]^+^	[M+O-2H+H]^+^	hydroxylation-dehydrogenation
260.0269	260.02807	−4.50	[C_10_H_15_BrNO_2_]^+^	[M-C_8_H_8_O+H]^+^	*N*-desalkylation
258.0124	258.01242	−0.08	[C_10_H_13_BrNO_2_]^+^	[M-C_8_H_8_O-2H+H]^+^	*N*-desalkylation-dehydrogenation
366.0680	366.06993	−5.27	[C_17_H_21_BrNO_3_]^+^	[M-CH_2_+H]^+^	*O*-desmethylation
352.0524	352.05428	−5.34	[C_16_H_19_BrNO_3_]^+^	[M-2CH_2_+H]^+^	bis-*O*,*O*-desmethylation
408.0808	408.08050	0.74	[C_19_H_23_BrNO_4_]^+^	[M+CO+H]^+^	carbonylation (formylation)
440.0710	440.07033	1.52	[C_19_H_23_BrNO_6_]^+^	[M+CO+2O+H]^+^	carbonylation (formylation)-bis-hydroxylation

^a^ Computed by ChemCalc (https://www.chemcalc.org/, accessed on 9 September 2025). ^b^ Computed by mass error calculator (https://warwick.ac.uk/fac/sci/chemistry/research/barrow/barrowgroup/calculators/mass_errors/, accessed on 9 September 2025).

**Table 3:** An overview of all relevant compounds identified in a post-mortem urine sample (Case No. 1) with HPLC-(ESI)-Q-TOF-MS analysis. 25B-NBOMe was detected in a gastric contents sample (Case No. 2). These results are consistent with the data shown in Figure 4.

Retention Time [min]	Measured *m*/*z* ^a^	Theoretical *m*/*z* ^b^	Relative Mass Accuracy [ppm]	Ion Formula	Modification	Metabolic Reaction
9.45	380.0876	380.08558	5.31	[C_18_H_23_BrNO_3_]^+^	[M+H]^+^	(parent compound)
8.80	366.0710	366.06993	2.92	[C_17_H_21_BrNO_3_]^+^	[M-CH_2_+H]^+^	*O*-desmethylation/phase I
8.72	446.0281	446.02675	3.03	[C_17_H_21_BrNO_6_S]^+^	[M-CH_2_+SO_3_+H]^+^	*O*-desmethylation and sulfation/phase II
7.91	542.1024	542.10202	0.70	[C_23_H_29_BrNO_9_]^+^	[M-CH_2_+C_6_H_8_O_6_+H]^+^	*O*-desmethylation and glucuronidation/phase II

^a^ Computed by ChemCalc (https://www.chemcalc.org/, accessed on 9 September 2025). ^b^ Computed by mass error calculator (https://warwick.ac.uk/fac/sci/chemistry/research/barrow/barrowgroup/calculators/mass_errors/, accessed on 9 September 2025).

**Table 4:** MS/MS fragments of 25B-NBOMe metabolites identified in real biological material.

*m*/*z* of Parent Ion	*m*/*z* of Product Ions	CE [V]
366	243, 201, 179, 121, 55	24.6
446	229, 121, 93, 91, 55	27.8
542	366, 275, 184, 121, 91	31.7

## Data Availability

The datasets generated during and/or analyzed during the current study are available from the corresponding author upon reasonable request.
